# MPPTM: A Bio-Inspired Approach for Online Path Planning and High-Accuracy Tracking of UAVs

**DOI:** 10.3389/fnbot.2021.798428

**Published:** 2022-02-11

**Authors:** Xin Yi, Anmin Zhu, S. X. Yang

**Affiliations:** ^1^Research Institute of Intelligence Technology and System Integration, College of Computer Science and Software Engineering, Shenzhen University, Shenzhen, China; ^2^Advanced Robotics and Intelligent Systems (ARIS) Laboratory, School of Engineering, University of Guelph, Guelph, ON, Canada

**Keywords:** multi-robot system, path planning, neural dynamics, path tracking, neural network

## Abstract

The path planning and tracking problem of the multi-robot system (MRS) has always been a research hotspot and applied in various fields. In this article, a novel multi-robot path planning and tracking model (MPPTM) is proposed, which can carry out online path planning and tracking problem for multiple mobile robots. It considers many issues during this process, such as collision avoidance, and robot failure. The proposed approach consists of three parts: a neural dynamic path planner, a hyperbolic tangent path optimizer, and an error-driven path tracker. Assisted by Ultra-wideband positioning system, the proposed MPPTM is a low-cost solution for online path planning and high-accurate tracking of MRS in practical environments. In the proposed MPPTM, the proposed path planner has good time performance, and the proposed path optimizer improves tracking accuracy. The effectiveness, feasibility, and better performance of the proposed model are demonstrated by real experiments and comparative simulations.

## 1. Introduction

As the development of disciplines and technologies, robotics always involves numerous disciplines. It covers many aspects from control, mechanics, electronics to communication, computer science, materials, and so forth. Robotics has also developed from a simple single robot system (SRS) to a complex multi-robot system (MRS). For dealing with complex problems, MRS has more advantages than SRS. Large numbers of researches state that the cooperation of MRS has been applied to more practical fields, such as services (Morita et al., 2018; Krizmancic et al., 2020), therapy (Ali et al., 2019; Mehmood et al., 2019), rescue (Queralta et al., 2020), training (Xu and Tang, 2021), and so on.

Path planning and tracking problem of MRS has always been a research hotspot and applied in various fields, including delivery (Chen et al., 2021), monitoring (Silic and Mohseni, 2019; Koutras et al., 2020), task assignment (Chen and Zhu, 2019; Wang et al., 2020b), target tracking (Zhou et al., 2018), and so on. In order to solve this problem, many kinds of research can be divided into three aspects: task-level, control-level, and task-control-level (Zeng et al., 2015; Rub́ı et al., 2019). The task-level research focuses on finding the optimal solution to the problem without considering the hardware conditions, which is top-down. It includes path planning of MRS and task assignment of MRS. The control-level research needs to consider the hardware of MRS and the realization of the solution, which is down-top. Tracking the target or path with high accuracy is one of these kinds of research. The task-control-level research combines task-level and control-level, and the path planning and tracking problem is one of this kind of research.

In the task-level research of MRS, path planning and task assignment are the two mainstreams. Compared with task assignments, path planning is more focused on the time-space continuity and process. For example, these studies (Yi et al., 2017; Dai et al., 2019; Ali et al., 2020; Dong et al., 2020; Han and Yu, 2020; Wang et al., 2020a) only consider task-level without considering control-level details. Combining with deep learning, (Wang et al., 2020a) proposed a neural RRT^*^ for path planning of MRS, but it needs a lot of processed data for training before path planning (Wang et al., 2020a). Han et al. used database heuristics for fast near-optimal path planning of MRS, it can carry out efficient path planning, but its applicable scene is only based on grid environment (Han and Yu, 2020). Yi et al. proposed a bio-inspired approach to plan the path of robots in 3-D environments. Also, it can make real-time path planning but can not avoid obstacles (Yi et al., 2017). Dong et al. proposed a path planning method of UAVs in the 3-D environment for the inspection of transmission lines. But it is only made available for a single target, not for multiple targets (Dong et al., 2020).

Path tracking for MRS is solved by control-level algorithms. These algorithms tend to reduce error during the tracking process while considering the difference of robot hardware in MRS. Ma proposed cooperative tracking of MRS with circular formation, but it can not track multi-target (Ma, 2020). Yu et al. proposed a formation tracking method based on an adaptive neural network, but it just makes MRS formate to track a single target (Yu et al., 2018). Zhou et al. presented a resilient tracking method for MRS. It is suitable for patrol and monitoring in the area but can not track the immovable target (Zhou et al., 2018).

Both task-level and control-level studies are very limited in practical application. Therefore, some studies focusing on both task-level and control-level have occurred.

Park et al. proposed a distributed approach combing alternating direction method of multipliers (ADMM) to non-myopic path planning for multi-target tracking, but it can not avoid obstacles in the environment (Park et al., 2019). Yordanova et al. proposed a path planning and tracking method for the area coverage of autonomous underwater vehicles, but in essence, the method is still only for the 2-D environment without obstacles (Yordanova and Gips, 2020). Penin et al. proposed a vision-based path planning and target tracking method for UAVs (Penin et al., 2018). It can deal with collision avoidance and obstacle avoidance, but its accuracy of vision-based positioning is still questionable for indoor environments.

Compared with these studies (Penin et al., 2018; Park et al., 2019; Yordanova and Gips, 2020; Yu et al., 2020), there are few studies for online path planning and tracking of MRS. During online path planning and tracking of MRS, the proposed model needs to plan the trajectories easy to track in real-time, which deals with dynamic environments and accidents, such as robot fault, moving obstacles, and so on. Online path planning and tracking need to solve the following three problems. (1) How can path planning meet the requirements of real-time; (2) How can the planned paths be transformed into trajectories easy to track; and (3) How to efficiently organize related processes?

In this study, a novel model named MPPTM (multi-robot path planning and tracking model) is proposed for online path planning and high-accuracy tracking of MRS. It does not depend on the sensors of the individual robot in MRS by using an Ultra-wideband (UWB) positioning system. Therefore, the proposed approach is a low-cost solution for warehouse or factory environments. The proposed model has the following innovations.

1. The proposed model uses superscalar pipelining mode to organize these processes more efficiently. Therefore, the process of path tracking does not need to wait until the end of path planning.
2. Compared with traditional path planners, the proposed neural dynamic path planner has better time performance.
3. In our proposed model, the hyperbolic tangent path optimizer bridges the planned paths and the trajectories easy to track, and it reduces the tracking error of MRS.

The remainder of the article is organized as follows. In section 2, the components and framework of the proposed model are introduced in detail. The experiments for MRS in a 3-D environment are present in section 3. Some further discussions about the comparative studies are given in section 4. Finally, the conclusion and future study are presented in section 5.

## 2. Proposed Approach

The proposed MPPTM is described in detail in this section. It is applicable for not only 2-D but also 3-D environments. It combines the task-level and control-level, which can deal with the path planning of MRS, and cope with the path tracking of MRS.

The proposed MPPTM mainly integrates three parts, including the path planner, the path tracker, and the path optimizer. The path planner is responsible for dealing with the online path planning of MRS at the task-level. During the path planning of MRS, obstacles avoidance, collision avoidance, and other robot accidents are considered. The path tracker is used to cope with the path tracking of MRS. During the path tracking of MRS, reducing tracking error and compatibility with different hardware are considered. The path optimizer is used to bridge the gap between the path planner and the path tracker, which makes the planned path to be easier tracked. It can process the online path planning and path tracking for MRS in complex environments.

**Figure 1:**
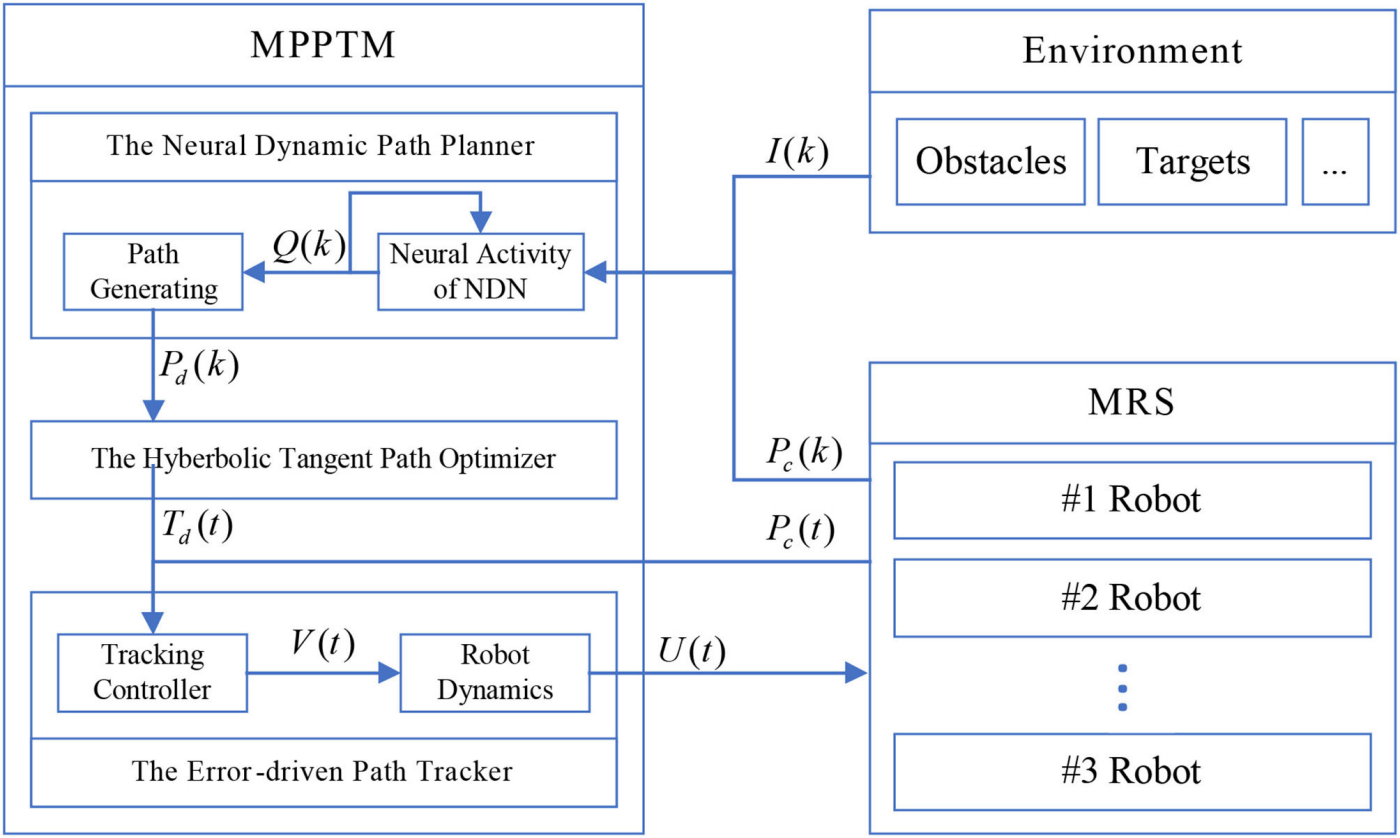
Overall schematic diagram of multi-robot path planning and tracking model (MPPTM) with environments.

The framework of MPPTM with environments is shown in Figure 1. The process of path planning and tracking, the cooperation mechanism between the path planner and the path tracker are given in the framework of MPPTM. In Figure 1, *k* and *t* are the iterations of the planner and the iteration of the tracker, respectively. For the path planner, *I*(*k*) represents the environmental information, and *P*_*c*_(*k*) represents the current posture of the MRS. Both are used to generate the desired posture *P*_*d*_(*k*) through *Q*(*k*). Through the path optimizer, the desired posture *P*_*d*_(*k*) is transferred into the desired trajectory *T*_*d*_(*t*) for the path tracker to track. In the path tracker, the desired posture *T*_*d*_(*k*) and current posture *P*_*c*_(*t*) of MRS are used to generate the desired velocity *V*(*t*) by path tracker for MRS. Additionally, then it is converted into the desired motor speed *U*(*t*) by robot dynamics. In the MRS, each robot tries to achieve the desired motor speed *U*(*t*) to move.

Usually, there are two different modes to deal with the path planning and tracking of MRS, which are superscalar pipelining mode and traditional mode.

In traditional mode, path tracking must wait until all path planning and optimization are completed. This mechanism handles these processes serially, which can save computing resources. However, it is only suitable for path planning and tracking of MRS in static environments.

**Figure 2:**
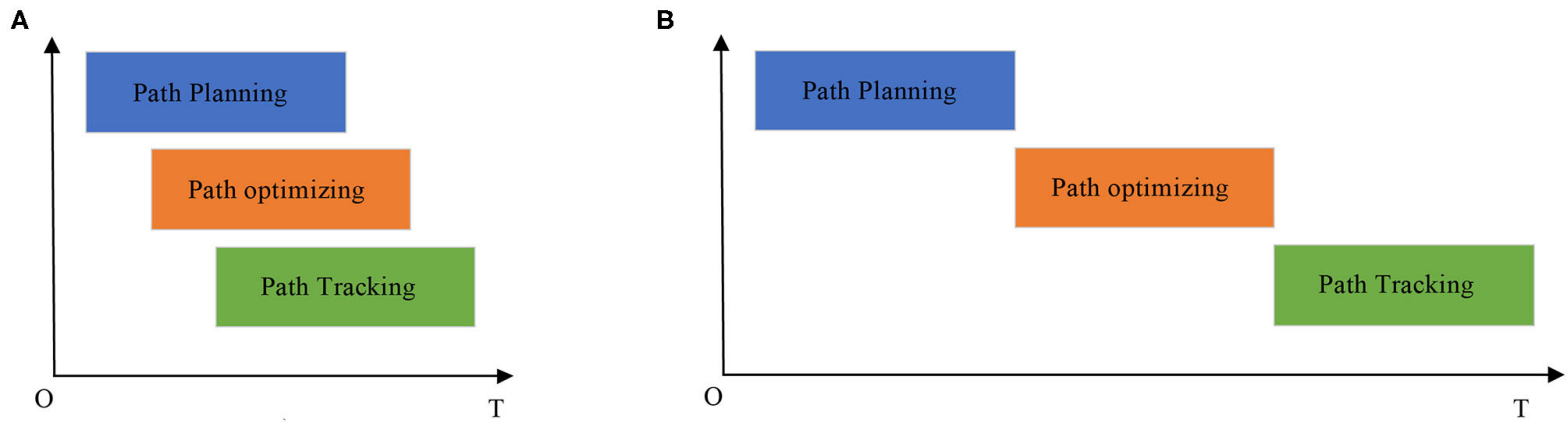
Two different operation modes in MPPTM. **(A)** The superscalar pipelining mode. **(B)** The traditional mode.

Due to the time performance of the proposed path planner, the proposed MPPTM applies superscalar pipelining mode to deal with these processes, such as path planning, path optimizing, and path tracking, as shown in Figure 2.

In superscalar pipelining mode, part of the planned path is used to be optimized by the path optimizer, and tracked by the path tracker. Path tracking does not have to wait for all path planning and optimization to be completed. This mechanism parallels these processes to some extent, which can be suitable for online path planning and tracking of MRS in dynamic environments.

The proposed MPPTM is introduced in detail by three following parts: (A) The neural dynamic path planner; (B) The error-driven path tracker; and (C) The hyperbolic tangent path optimizer.

### 2.1. The Improved Neural Dynamic Path Planner

As an important part of MPPTM, the neural dynamic path planner is used to plan paths for MRS. It is based on neural dynamics and has the advantages and characteristics of the biological neural system. The neural dynamic path planner for 3-D environments is introduced in the section, and the proposed path planner for 2-D environments is similar to this. The neural dynamic path planner includes three following parts.

#### 2.1.1. The Neural Dynamic Network (NDN)

**Figure 3:**
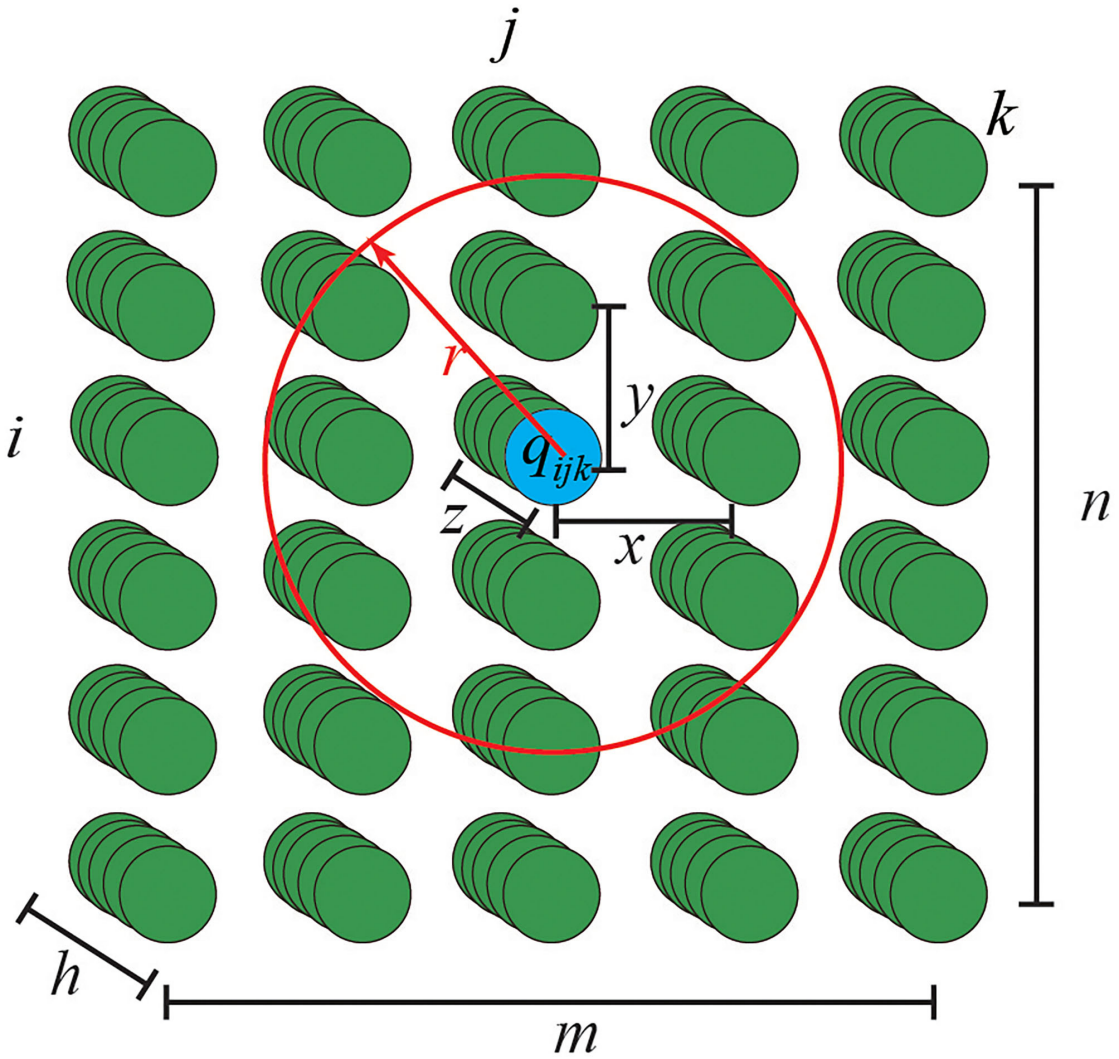
The neural dynamic network (NDN) describing 3-D environments.

In the neural dynamic path planner, the NDN is used to real-time describe the environment where MRS is located. In NDN, the distance between any two adjacent neurons is equal, its distance is 1, and any two adjacent neurons are also connected to each other. The structure of NDN describing the 3-D environment is as shown in Figure 3.

The activity of neuron *q*_*ijk*_ is in the *i*th row, the *j*th column, and the *k*th page of the NDN, which describes the environment that it maps. The activities of NDN *Q* describing 3-D environments is a 3-D matrix defined by Equation (1).


(1) $$\begin{array}{l} Q={\left[\begin{array}{c} \left[\begin{array}{ccc} q_{111} & \cdots & q_{1n1}\\ \vdots & \ddots & \vdots \\ q_{m11} & \cdots & q_{mn1} \end{array}\right]\\ \vdots \\ \left[\begin{array}{ccc} q_{11h} & \cdots & q_{1nh}\\ \vdots & \ddots & \vdots \\ q_{m1h} & \cdots & q_{mnh} \end{array}\right] \end{array}\right]}_{m\times n\times h} \end{array}$$

In Figure 3, *r* is the radius of the range that neurons can affect, *x* is horizontal offset, *y* is vertical offset, *z* is longitudinal offset, and *x, y, z* are integers. The radius of the search sphere *r* directly affects the computation performance and the accuracy of path planning. If radius *r* is set too large, the planned path will pass through the obstacles in the environment. In NDN, the activity of each neuron *q* represents the environment where it maps. Therefore, the activities of NDN *Q* describe the whole environment.

#### 2.1.2. The Improved Neural Activity Algorithm

This algorithm states the activity of neural signals in NDN. Through multiple iterations of neural activity, the activities of NDN *Q* tend to be stable, and all kinds of signals have been fully spread in NDN. The neural activity of NDN is the core part of the proposed path planner, and it is also the most time-consuming process. Therefore, we proposed an improved neural activity algorithm of NDN, and it is defined by Equation (2).


(2) $$\begin{array}{l} \frac{dQ}{dt}=-KQ+\left(D-Q\right)\left({\left[I\right]}^{+}+{\left[F\left(x,y,z\right)\right]}^{+}\right)\\ \;-\left(J+Q\right)\left({\left[I\right]}^{-}+{\left[F\left(x,y,z\right)\right]}^{-}\right) \end{array}$$

Three parameters, *K, D, J*, are decay rate, upper bound, and lower bound, respectively, in the dynamic equation of neural activity. Meanwhile, two operators, [*a*]^+^ and [*b*]^−^, obtain, respectively, *max*{*a*, 0} and *max*{−*a*, 0}. The function *F*(*x, y, z*) is the weighted sum of the shifting matrix *Q* with these offsets *x, y, z*, and it is defined by Equation (3).


(3) $$\begin{array}{l} F\left(x,y,z\right)=\underset{xyz}{\sum }w_{xyz}shift\left(Q,x,y,z\right)\sqrt{x^{2}+y^{2}+z^{2}}\leq r \end{array}$$

where *shift*(*Q, x, y, z*) shifts the elements of matrix *Q* with the *x* rows, the *y* columns, and the *z* pages, but it satisfies the condition $\sqrt{x^{2}+y^{2}+z^{2}}\leq r$. This weight *w*_*xyx*_ is defined by Equation (4).


(4) $$\begin{array}{l} w_{xyz}=\frac{u}{\sqrt{x^{2}+y^{2}+z^{2}}}\sqrt{x^{2}+y^{2}+z^{2}}\leq r \end{array}$$

where *w*_*xyz*_ is the connection weight when the horizontal offset is *x*, the vertical offset is *y*, and longitudinal offset is *z* in NDN, *u* is the positive parameter and represents the intensity of the connection.

In Equation (2), environmental information *I* is a 3-D matrix with the same size as *Q*, its element is defined as Equation (5). *Ex* and *In* are positive parameters, which represent the intensity of excitatory nerve signal and inhibitory nerve signal, respectively, in NDN.


(5) $$i_{ijk=}\{\begin{array}{c} Ex\\ -In\\ 0 \end{array}\begin{array}{c} \text{The neuron maps target}\\ \text{The neuron maps robot}\\ \text{Others} \end{array}$$

#### 2.1.3. The Path Generation for 3-D Environments

After the multiple iterations of neural activity, the activities of NDN *Q* are used to generate the next position during the path planning of MRS. The next position of the *i*_*th*_ robot *P*^*i*^(*k* + 1) is defined as Equation (6).


(6) $$\begin{array}{l} P^{i}\left(k+1\right)⇐q_{abc}=max\left\{q_{efg}|\begin{array}{c} 0<||q_{efg}-q_{ijk}|| \end{array}<r\right\} \end{array}$$

Assume that the current position of the *i*_*th*_ robot is mapped by the neuron *q*_*ijk*_, *q*_*efg*_ is the set of neurons in the affected range of the neuron *q*_*ijk*_. In the set *q*_*efg*_, the maximum activity of the neuron *q*_*abc*_ is selected as the next position *P*^*i*^(*k* + 1) of the *i*_*th*_ robot during path planning.

### 2.2. The Error-Driven Path Tracker

The proposed path tracker uses error driven method to track the path planned by the proposed path planner. The error-driven path tracker is capable of being compatible with a variety of controllers and the hardware of MRS. In order to better test the performance of the proposed MPPTM in 3-D environments, a path tracker with PID controller for quadrotor is given briefly in the article, which is used in the following experiments.

**Figure 4:**
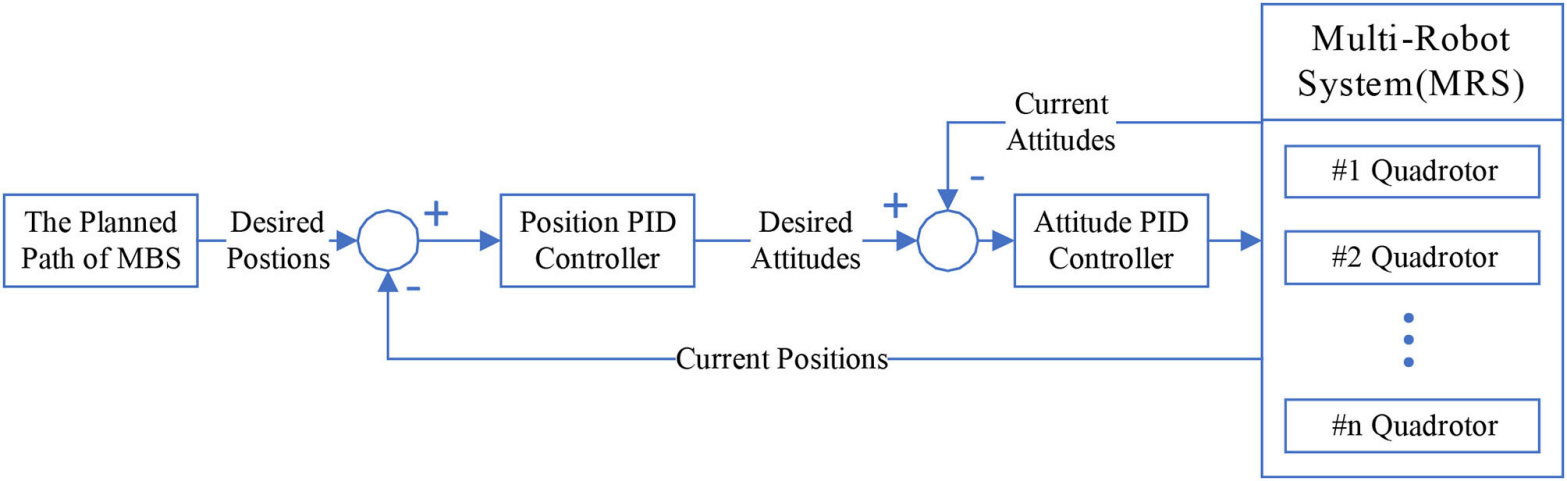
The proposed path tracker for quadrotors in 3-D environments.

Quadrotor, a helicopter with four rotors, is a small unmanned aerial vehicle (UAV). Additionally, it is used as an individual MRS in 3-D environments. Two control loops with PID control are used in the path tracker of MRS for path tracking in 3-D environments. The system architecture of the proposed path tracker for 3-D tracking with PID control is shown in Figure 4.

### 2.3. The Hyperbolic Tangent Path Optimizer

The path optimizer bridges the gap between the path planner with the path tracker, it translates the task-level paths planned by the path planner into the control-level paths for the path tracker. During this process, the task-level paths *P*^*i*^(*k*) with low frequency should be transformed into the control-level trajectory *T*^*i*^(*t*) with high frequency, which can make the planned paths easy to track for MRS. The planned paths are given by the path planner are defined as Equation (7), where *Paths*^*i*^(γ) is the continuous planned path of the *i*_*th*_ robot in MRS, *k* is the iteration of the path planner, and *R* is the number of robots in MRS.


(7) $$\begin{array}{l} P^{i}(k)\in \{Paths^{i}(\gamma )|\begin{array}{cc} i=1,2\ldots ,R & \gamma \geq 0 \end{array}\}\\ \;\begin{array}{cc} k=1,2\ldots ,n & i=1,2\ldots ,R \end{array} \end{array}$$

The control-level trajectory $T_{os}^{i}\left(t\right)$ given by the path optimizer with original sampling from *P*^*i*^(*k*) can be obtain by Equation (8), where the operator ⌊*a*⌋^*b*^ obtains the largest element in set *b* and less than or equal to *a*, and *w* is the sampling frequency.


(8) $$\{\begin{array}{c} a={\left\lfloor t/w\right\rfloor }^{k}\\ T_{os}^{i}(t)=[P^{i}(a+1)-P^{i}(a)]\times \frac{t\%w}{w}i=1,2\ldots R \end{array}$$

In order to improve the tracking performance of MRS, an improved sampling method is proposed in the article. *P*^*i*^(τ) is the set of elements in set *P*^*i*^(*k*) whose elements are not differentiable on the paths of MRS, which can describe as Equation (9).


(9) $$\begin{array}{l} P^{i}(\tau )\in \left\{P^{i}(k)|\begin{array}{c} \underset{\Delta \delta \to 0^{+}}{\lim}{\dot{P}}^{i}(k+\Delta \delta )\neq \underset{\Delta \delta \to 0^{-}}{\lim}{\dot{P}}^{i}(k+\Delta \delta )\\ k\in 1,2\ldots ,n \end{array}\right\}\\ \;i\in 1,2\ldots ,R \end{array}$$

The trajectory $T_{hts}^{i}\left(t\right)$ given by the path optimizer with hyperbolic tangent sampling from *P*^*i*^(τ) can be obtain by Equation (10), where the operator ⌈*a*⌉^*b*^ obtains the smallest element in set *b* and more than or equal to *a*, *w* is the sampling frequency.


(10) $$\{\begin{array}{c} a={\left\lceil t/w\right\rceil }^{\tau }\\ b={\left\lfloor t/w\right\rfloor }^{\tau }\\ T_{hts}^{i}(t)=[P^{i}(a)-P^{i}(b)]\times f(\frac{t-w\times b}{w\times (a-b)})\;i=1,2\ldots R \end{array}$$

where *f*(*x*) is the hyperbolic tangent function defined as Equation (11). Optimal trajectory with hyperbolic tangent sampling $T_{hts}^{i}$ is generated by optimizing the path *Paths*^*i*^(γ) according to the referenced velocity.


(11) $$\begin{array}{l} f\left(x\right)=0.5\times \left(\frac{e^{2x+1}-1}{e^{2x+1}+1}+1\right) \end{array}$$

Compared with the path planner with original sampling, the one with hyperbolic tangent sampling can give the trajectory which is easier to track for MRS, which is demonstrated in the following experiment.

## 3. Experiments

For demonstrating the feasibility of the proposed MPPTM, online path planning and tracking experiments for multi-UAV are designed in this section. However, it should be noted that the proposed MPPTM is suitable for online path planning and tracking of MRS not only in 2-D environments but also in 3-D environments.

### 3.1. Experimental Preparations

**Figure 5:**
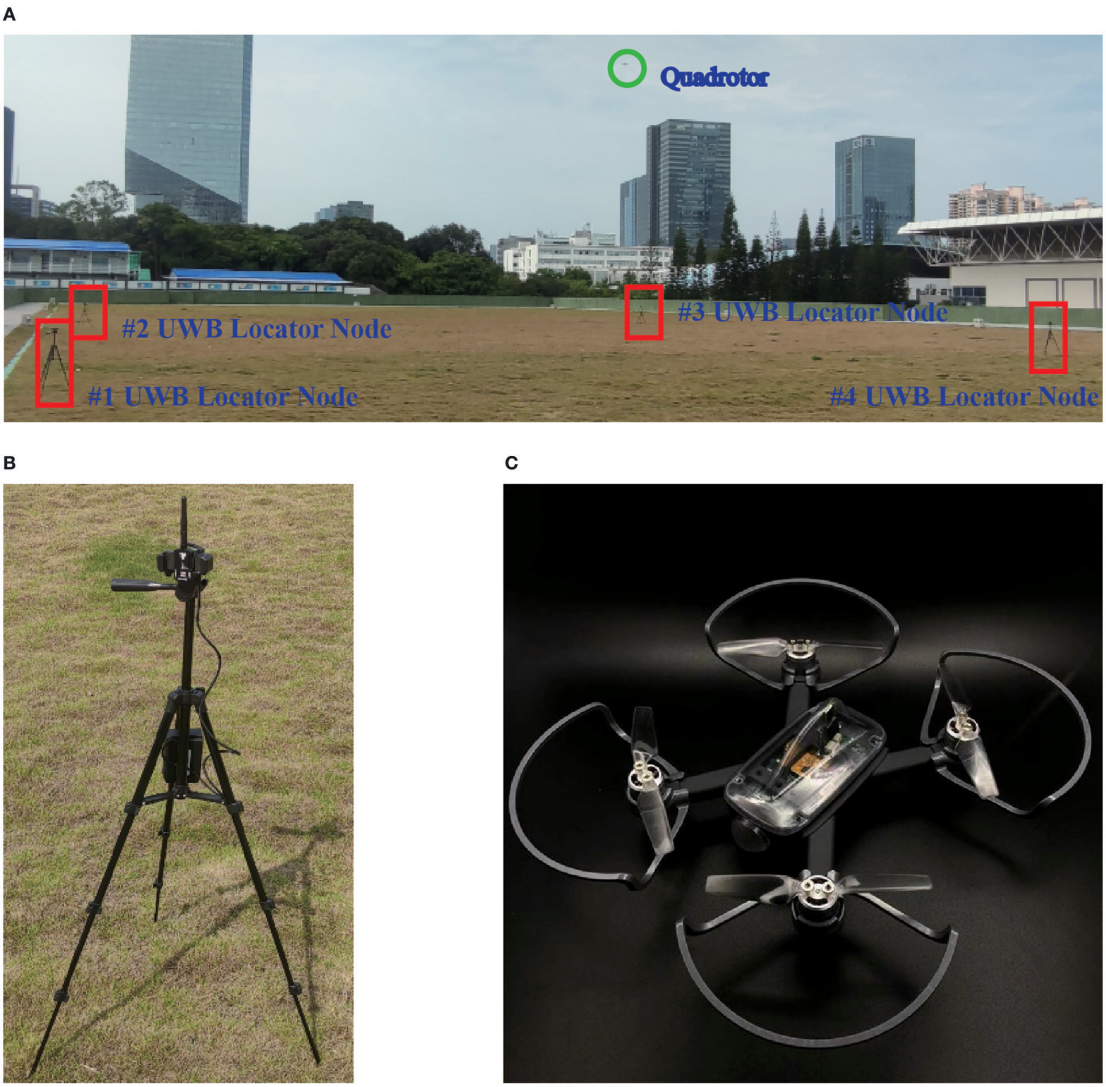
The experimental preparations for online path planning and tracking. **(A)** The experimental site for online path planning and tracking of multi-UAVs. **(B)** The UWB locator node. **(C)** A micro quadrotor with UWB tag.

In order to accurately locate UAV groups in 3-D environments, the UWB positioning system is used, as shown in Figure 5. At least four UWB locator nodes are used to locate UAV groups in 3-D environments, and the distance between two adjacent nodes is 15*m*. A micro quadrotor produced by Zeronetech is used as an individual of the UAV group in this experiment, and it has a built-in UWB tag and wireless communication module.

The laptop with R5800u CPU and 32GB ROM is responsible for collecting the location data of UAVs *via* the UWB positioning system, recoding the flight data of UAVs, and controlling the fight of UAVs *via* WiFi communication. The system is implemented by Matlab and C++, the proposed path planner is coded by Matlab, the proposed path optimizer and the proposed path tracker are coded by C++. The obstacles in 3-D environments are realized by marking the environmental information *I*(*k*) in the proposed path planner.

### 3.2. Online Path Planning and Tracking for 3-D Environments

The size of the outdoor environment used for testing is 10 m×10 m×10 m, which is mapped by the NDN with size 50×50×50 in the neural dynamic path planner. In the proposed path planner, we set *K* to 50, *D* to 5, *J* to 3, *u* to 0.3, *r* to 2, *Ex* to 50, and *In* to 5. In the proposed path optimizer, hyperbolic tangent sampling is used at the condition *w* = 100. The initial positions of targets and quadrotors are randomly located. The initial sizes and initial positions of obstacles are randomly generated to mark the environmental information *I*(*k*).

**Figure 6:**
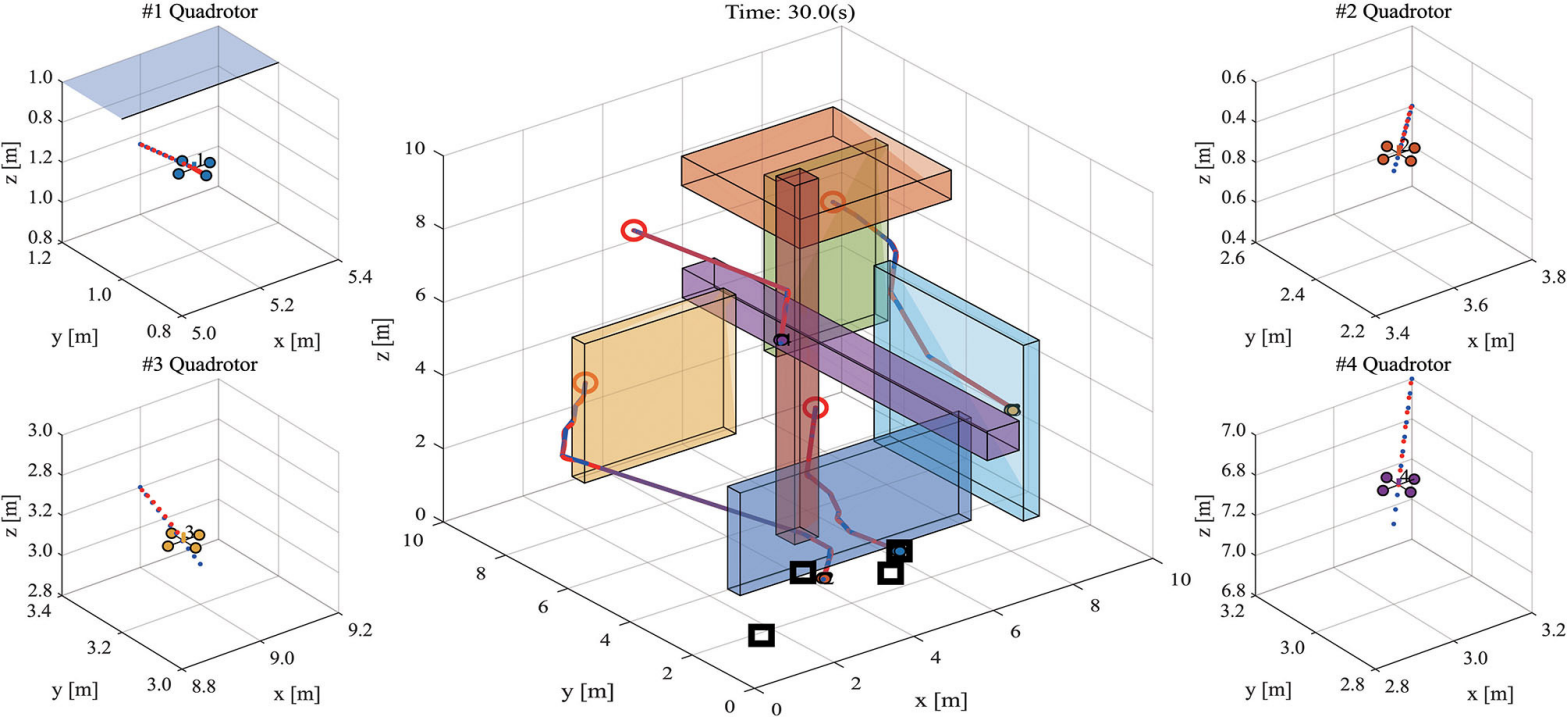
The snapshot of path planning and tracking for UAVs in 3-D environments at 30 s. (Red circles and black squares present the initial positions of robots and targets respectively on the main diagram. Four sub diagrams zoom in the main diagram and show the current flight states of quadrotors. The red dot line presents the real path and the blue dot line presents the referenced path).

**Figure 7:**
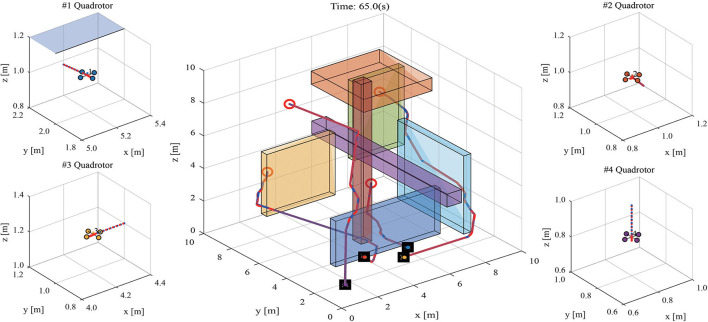
The snapshot of path planning and tracking for UAVs in 3-D environments at 65 s. (The meaning of markers is the same as that in Figure 6).

The snapshot of path planning and tracking at 30 s is shown in Figure 6, and the 1th quadrotor has finished capturing the target. Figure 7 shows the snapshot of path planning and tracking at 65 s, where all quadrotors have captured targets.

**Figure 8:**
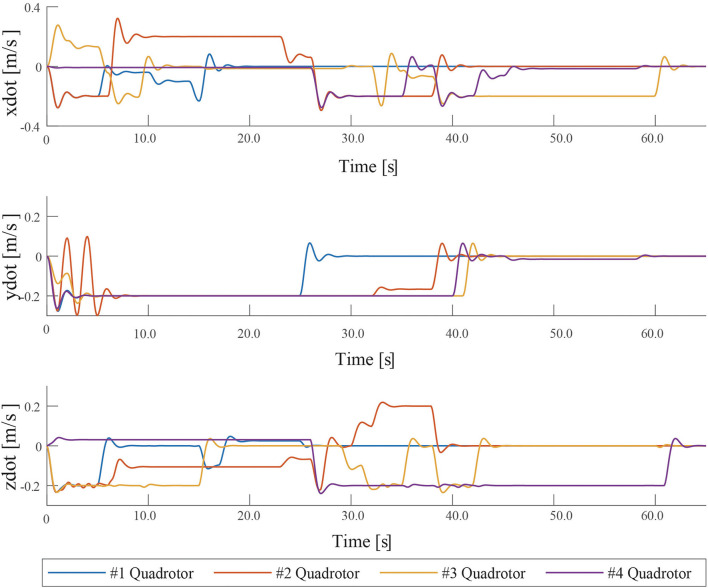
The snapshot of path planning and tracking for UAVs in 3-D environments at 65 s.

The velocities of quadrotors in *X, Y, Z* directions are recoded and shown in Figure 8. The experimental result shows that all quadrotors can avoid collision and obstacles, and capture targets. The experimental result demonstrates that MPPTM is capable of online path planning and tracking of MRS in 3-D complex environments.

### 3.3. Online Path Planning and Tracking With Robot Fault

This experiment uses the same parameters and the same environment from the experiment in section 3.2. But in the online path planning and tracking, there are two quadrotors that have a certain probability 0.05 of failure during the process. Additionally, MPPTM needs to allocate these two targets to the other two quadrotors during the process.

**Figure 9:**
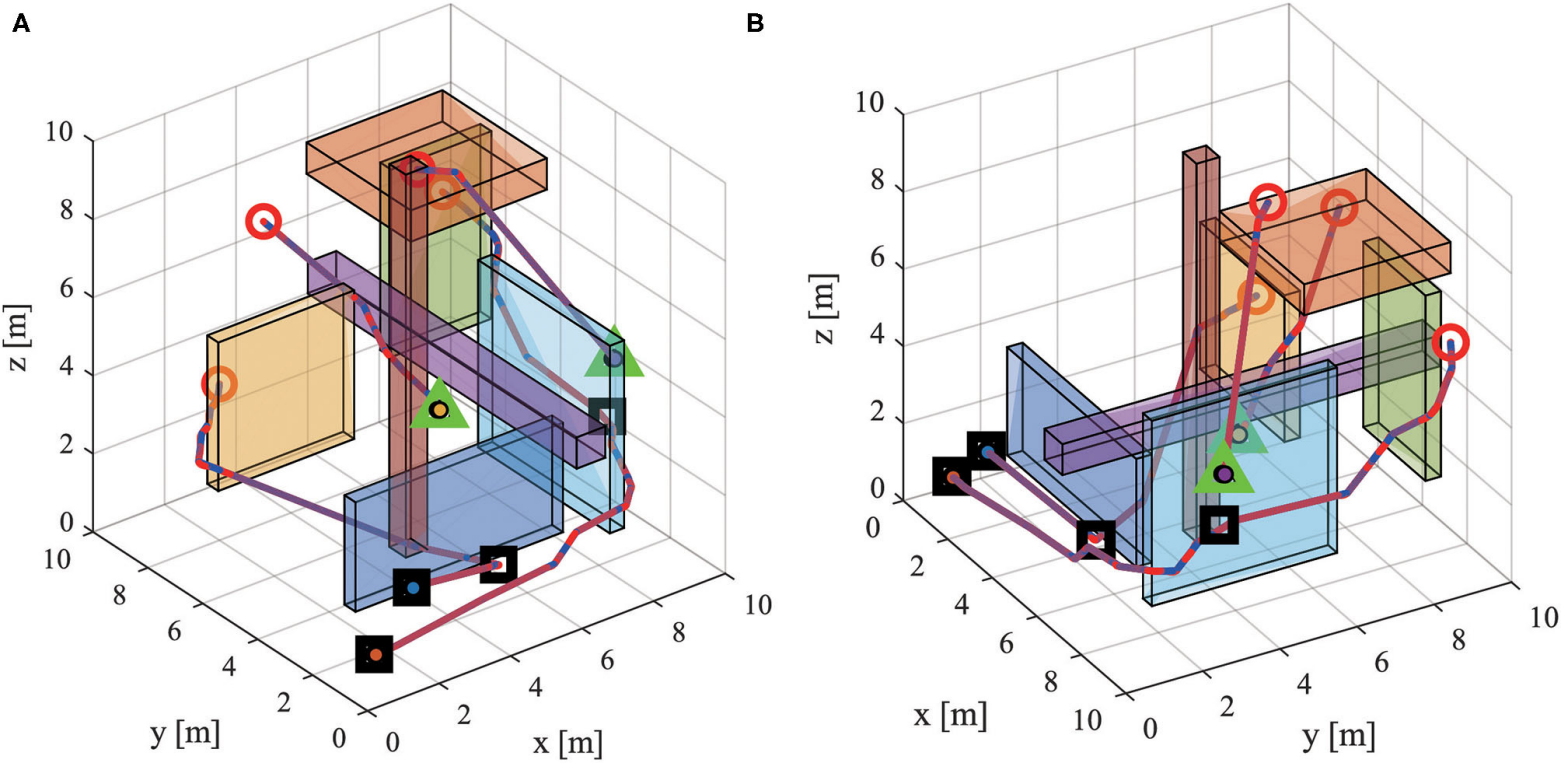
The snapshot of path planning and tracking with quadrotor fault in 3-D environments. **(A)** Observation from view (-38, 30). **(B)** Observation from view (60, 30). (Green triangle represents the position of quadrotor fault. The meaning of other markers are the same as that in Figure 6).

As shown in Figure 9, after two quadrotors break down, the other quadrotors have also successfully completed four targets capturing. Therefore, the Experimental result indicates that MPPTM can deal with robot fault during online path planning and tracking, and it can reassign targets timely after robot fault.

### 3.4. Online Path Planning and Tracking With Dynamic Obstacles

**Figure 10:**
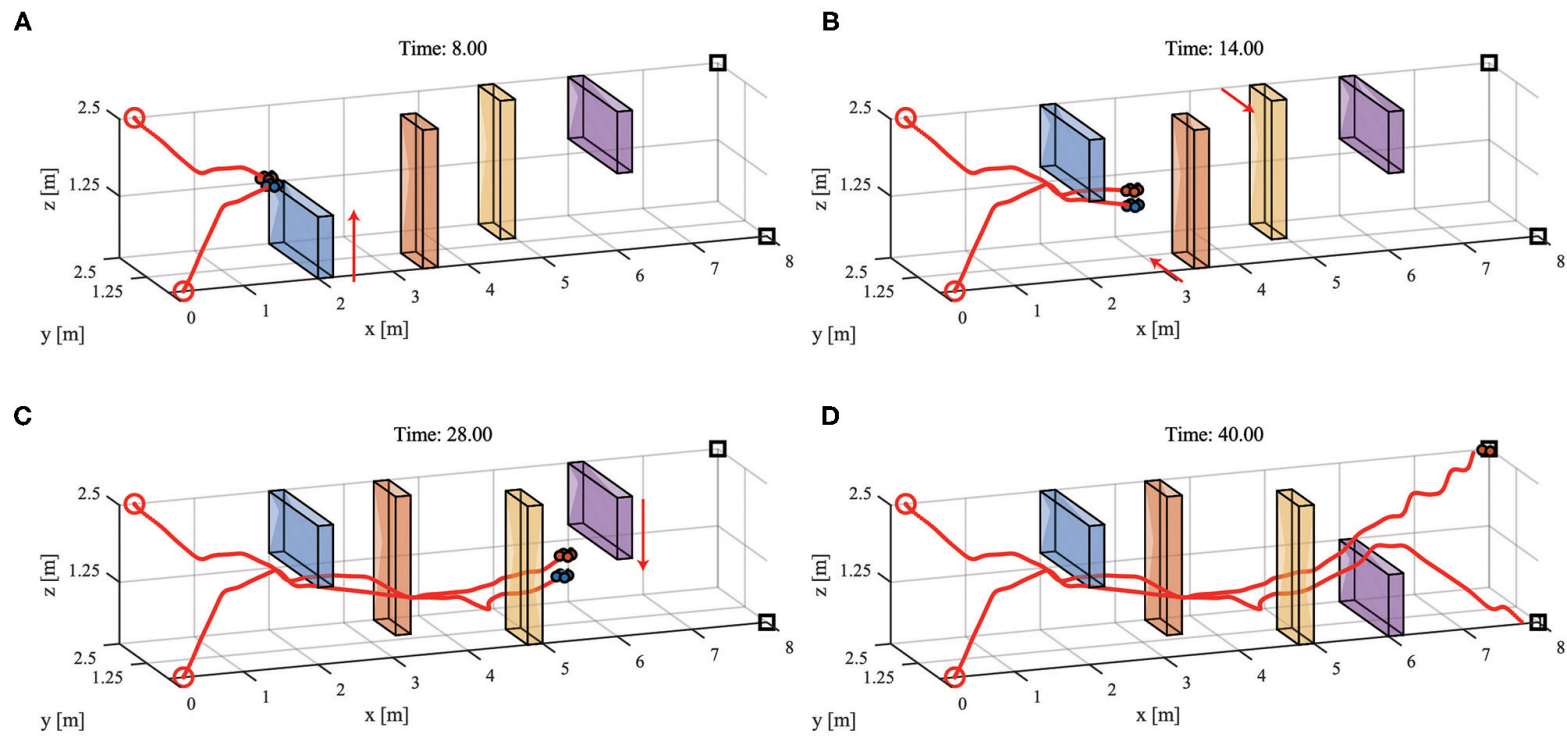
The path planning and tracking with dynamic obstacles in 3-D environments. **(A)** The snapshot at 8 s. **(B)** The snapshot at 14 s. **(C)** The snapshot at 28 s. **(D)** The snapshot at 40 s. (Red arrow represents the direction of obstacles movement. The meaning of other markers are the same as that in Figure 6).

This experiment is conducted in the 3-D workspace 8 m×2.5 m×2.5 m, and the experimental environment is mapped by the NDN with size 40 × 10 × 10. The experimental parameters are the same as those of the experiment in section 3.2. Obstacles in this experiment move at different times (8*s*, 14*s*, 28*s*) in the order shown in Figure 10. In order to better observe the experimental results, only two UAVs are used for the experiment. However, the proposed MPPTM can carry out online path planning and tracking for more robot individuals in an environment with dynamic obstacles.

Figure 10 shows that two quadrotors can avoid dynamic obstacles to capture targets during the path planning and tracking. The experimental result indicates that MPPTM carries out online path planning and tracking of MRS in an environment with dynamic obstacles.

## 4. Discussion

Several comparative experiments on the proposed path optimizer and the proposed path planner are given in this section.

### 4.1. The Performance of the Proposed Path Optimizer

**Figure 11:**
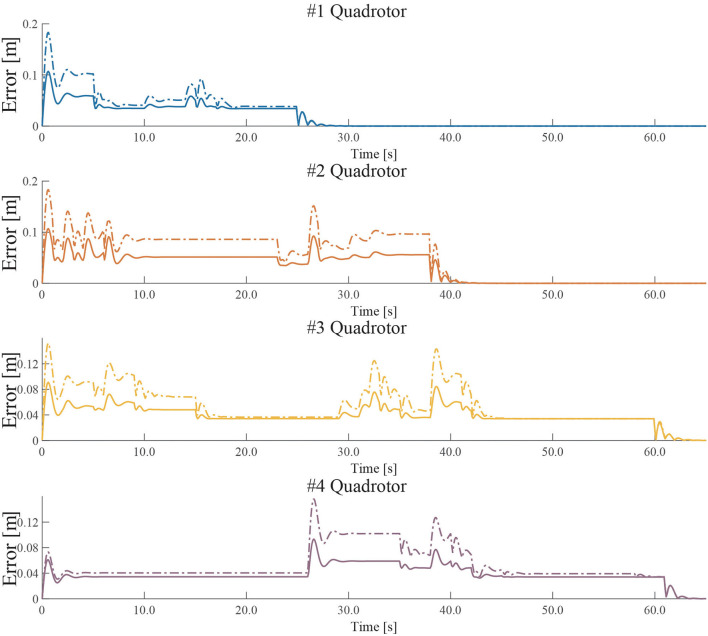
The tracking errors of quadrotors by using two different path optimizers. (The solid line represents the tracking error by using the hyperbolic tangent path optimizer, and the dashed line represents the tracking error by using the original path optimizer).

By using two different path optimizers, the actual paths and the desired paths are recoded in the experiment in section 3.2. The tracking error is the sum of the errors between the actual path and the desired path on the *X*, *Y*, and *Z* axes. The errors of the quadrotors are shown in Figure 11.

An indicator, defined as Equation (12), is given to measure the tracking error of UAVs during online path planning and tracking, where *R* is the number of quadrotors in UAVs, *error*_*i*_(*t*) is the error the *i*_*th*_ quadrotor, and *t* is the time.


(12) $$\begin{array}{l} E=\frac{1}{R}\overset{R}{\underset{i=1}{\sum }}\overset{+\infty }{\underset{0}{\int }}error_{i}\left(t\right)dt \end{array}$$

In order to eliminate the influence of a series of factors as far as possible, such as wind speed, battery status, and measurement error, we test 20 experiments and collect the data of tracking error. These data are shown in Table 1.

**Table 1:** The comparison of tracking error of unmanned aerial vehicle (UAVs).

**Case**	***Max*(*E*)**	***Min*(*E*)**	***Avg*(*E*)**
The original path optimizer	1.5669	1.3768	1.4797
The proposed path optimizer	**1.0453**	**0.9765**	**1.0278**

*Bold indicates that this indicator is the most superior to other cases*.

Table 1 shows that, compared with the original path optimizer, the proposed path optimizer can reduce the tracking error of UAVs during online path planning and tracking.

### 4.2. The Time Performance of the Proposed Path Planner

By using the proposed neural activity algorithm, the proposed path planner has better time performance than other approaches (Li et al., 2009; Yi and Zhu, 2013; Sun et al., 2019; Ni et al., 2020; Zhu et al., 2021). The comparative simulations are coded by Matlab, which is run on the PC with Intel i7-7700, 28GB ROM, and Win10 OS. The average time consumptions of single path planning by using different approaches and different sizes are shown in Table 2.

**Table 2:** The average time consumptions of different approaches (s).

**Case**	**Size**	**R=4**	**R=8**	**R=12**	**R=16**
Our approach	30X30	**0.1738**	**0.1831**	**0.1686**	**0.1826**
	50X50	**0.8563**	**0.8322**	**0.8647**	**0.8534**
Li et al. (2009)	30X30	3.5184	7.4610	9.8962	15.7595
	50X50	18.3248	37.6820	46.9013	74.6896
Yi and Zhu (2013)	30X30	0.9502	0.8306	0.9999	0.9882
	50X50	5.0008	3.9363	5.0498	4.9412
Sun et al. (2019)	30X30	2.8010	5.6704	6.8507	10.5158
	50X50	13.2122	25.7745	34.4256	52.0586
Ni et al. (2020)	30X30	2.563	2.7275	2.9789	3.4007
	50X50	12.0897	13.8657	15.0516	18.0413
Zhu et al. (2021)	30X30	2.2681	4.5916	5.5473	8.5152
	50X50	10.6986	20.871	27.8763	42.1546

*Bold indicates that this indicator is the most superior to other cases*.

Table 2 indicates that the time performance of the proposed path planner is better than other approaches, and it is insensitive to the number of robots in MRS. The proposed path planner has excellent time performance, which makes it very suitable for superscalar pipelining mode in the proposed path planner of MPPTM for online path planning and tracking in 3-D environments.

## 5. Conclusion

The proposed model, MPPTM, can deal with task-level and control-level problems for path planning and tracking of MRS in 3-D environments. During the online path planning and tracking, our proposed model only needs to obtain the position of MRS instead of relying on the complex sensor data of individual robots to plan paths. The cost of MRS equipped with such complex sensors is huge. Therefore, our proposed model, MPPTM, is a low-cost solution for the online path planning of MRS. Based on the UWB positioning system, MPPTM can carry out online path planning and high-accuracy path tracking for MRS in indoor or outdoor environments. It is suitable for application in manufacturing plants and industrial parks. Real experiments in this article demonstrate the applicability and effectiveness of the proposed model. In this model, the proposed path planner has excellent time performance to meet the requirement of superscalar pipelining mode. Meanwhile, the proposed path optimizer can guarantee the high-accuracy tracking of UAVs.

## Data Availability Statement

The original contributions presented in the study are included in the article/supplementary material, further inquiries can be directed to the corresponding author/s.

## Author Contributions

XY and AZ contributed to the conception of the study. XY performed the experiment, the data analyses and wrote the manuscript. AZ contributed significantly to analysis and manuscript preparation. SY helped perform the analysis with constructive discussions. All authors listed have made a direct and intellectual contribution to the study, and approved it for publication.

## Funding

This study was supported by National Natural Science Foundation of China (61273354) and Shenzhen stability support program for university (20200812112522002).

## Conflict of Interest

The authors declare that the research was conducted in the absence of any commercial or financial relationships that could be construed as a potential conflict of interest.

## Publisher's Note

All claims expressed in this article are solely those of the authors and do not necessarily represent those of their affiliated organizations, or those of the publisher, the editors and the reviewers. Any product that may be evaluated in this article, or claim that may be made by its manufacturer, is not guaranteed or endorsed by the publisher.
